# Polydatin enhances oxaliplatin-induced cell death by activating NOX5-ROS-mediated DNA damage and ER stress in colon cancer cells

**DOI:** 10.3389/fphar.2024.1532695

**Published:** 2025-01-09

**Authors:** Qi Zhao, Yan Zhang, Jieyu Liu, Peipei Chen, Annabeth Onga, Namki Cho, Ri Cui, Chenguo Zheng

**Affiliations:** ^1^ The Second Affiliated Hospital and Yuying Children’s Hospital of Wenzhou Medical University, Wenzhou, Zhejiang, China; ^2^ Cancer and Anticancer Drug Research Center, School of Pharmaceutical Sciences, Wenzhou Medical University, Wenzhou, Zhejiang, China; ^3^ College of Pharmacy and Research Institute of Drug Development, Chonnam National University, Gwangju, Republic of Korea

**Keywords:** colorectal cancer, polydatin, oxaliplatin, ROS, NAPDH oxidase 5, DNA damage

## Abstract

**Background:**

Polydatin (3,4′,5-trihydroxy-3-β-d-glucopyranoside, PD) is known for its antioxidant and anti-inflammatory properties. Oxaliplatin (OXA)-based chemotherapy is the first-line treatment for metastatic and recurrent colorectal cancer (CRC). However, the lack of selectivity for normal cells often results in side effects. Consequently, the search for anti-cancer components with high efficacy and low cytotoxicity has become a significant focus in recent years.

**Methods:**

The anti-tumor effects of PD, OXA or their combination were assessed by cell viability, colony formation, and wound-healing assays. Reactive oxygen species (ROS) generation was measured by flow cytometry and DNA damage was assessed by immunofluorescence assay. The relative gene and protein expressions were analyzed by quantitative real time-PCR (qRT-PCR) and Western blot assays. Molecular docking analysis predicted the interaction between PD and potential targets.

**Results:**

We found that PD exerted anti-CRC activity by promoting Nicotinamide Adenine Dinucleotide Phosphate (NADPH) oxidase 5 (NOX5)-mediated ROS production, activating the endoplasmic reticulum (ER) stress, and inducing DNA damage. Knocking down NOX5 attenuated the inhibition of proliferation and colony forming ability induced by PD in colon cancer cells and reversed the expression of C/EBP-homologous protein (CHOP) and activating transcription factor 4 (ATF4) proteins. In addition, combination of PD and OXA synergistically exerted anti-CRC activities by promoting DNA damage and activating ER stress signaling pathway.

**Conclusion:**

The combination of PD and OXA could be an effective treatment strategy for certain patients with CRC.

## 1 Introduction

Colorectal cancer (CRC) is the third most prevalent malignant neoplasm in the world with high morbidity and mortality (Siegel et al., 2024). Although the treatment outcome of CRC patients has improved through early screening and improved medical technology, the 5-year survival rate is still unsatisfactory (Siegel et al., 2023). Currently, clinical treatments for CRC include traditional surgery, radiotherapy, chemotherapy, targeted therapy and immunotherapy (Van der Jeught et al., 2018). Chemotherapy, a neoadjuvant or adjuvant therapy, is an irreplaceable treatment for patients with locally advanced CRC and liver metastases from CRC (Mcquade et al., 2017). However, chemotherapeutic agents often exhibit high cytotoxicity and frequent resistance, limiting their clinical application (Anand et al., 2023). Therefore, discovering new drugs that can selectively target carcinogenic cells and enhance anti-tumor activity, along with elucidating their mechanism of action, has significant social and scientific value.

Oxaliplatin (OXA) is a third-generation platinum-derived anti-cancer drug. In clinical practice, OXA is commonly used in combination with 5-fluorouracil (5-FU) and other drugs to treat CRC patients. However, its clinical application remains limited owing to drug resistance and intestinal toxicity (Benson et al., 2021; Chang et al., 2020; Xie et al., 2020). In recent years, increasing evidence suggests combining OXA with other drugs may enhance its anti-CRC activity. The combination of ursolic acid and OXA synergistically impeded the colon cancer cell proliferation and caused apoptosis both *in vitro* and *in vivo via* inhibition of mitogen-activated protein kinase (MAPK), PI3K/AKT and NF-κB signaling pathways (Shan et al., 2016). Dihydroartemisinin (DHA) enhanced cytotoxicity of OXA by activating ROS-mediated endoplasmic reticulum (ER) stress and c-Jun N-terminal Kinase (JNK) signaling pathways in colon cancer cells (Yu et al., 2022). In addition, the combination of piperlongumine and OXA promotes reactive oxygen species (ROS) production and synergistically exerts anti-tumor efficacy in colon cancer cells, providing an effective strategy and a new theoretical basis for reducing OXA -induced cytotoxicity and enhancing the therapeutic efficacy against CRC (Chen et al., 2019). Therefore, discovering new drugs that can safely improve the therapeutic effects of OXA is of paramount importance.

Polydatin (PD), the main active ingredient of the traditional Chinese medicine *Reynoutria japonica* Houtt., is a glycosylated derivative of resveratrol with antioxidant, anti-tumor, and anti-inflammatory properties (Peng et al., 2013; Zeng et al., 2016). Increasing evidences suggest that PD has anti-tumor effects on CRC (Wu et al., 2015). PD inhibits colon cancer cell proliferation by inducing oxidative stress and mitochondrial dysfunction, and preventing MAPK and PI3K/AKT transduction signaling. In addition, PD and 5-FU combination therapy significantly inhibited colon cancer cell proliferation, and alleviated 5-FU resistance by enhancing apoptosis through calcium endocytosis (Bae et al., 2021). PD has been reported to stimulate ROS to promote apoptosis in various cancer cells. PD-loaded poly (lactic-co-glycolic acid) (PLGA) nanoparticles (POL-PLGA-NPs) induced ROS-mediated oxidative stress to augment apoptosis (Vijayalakshmi et al., 2021). However, the role and mechanism of PD in the anti-CRC activity of OXA remain unclear. Tumor occurrence and development are strongly associated with the different degrees of cellular response to oxidative stress. Low levels of ROS tend to promote cell survival and proliferation, whereas high levels of ROS induce oxidative stress, leading to proliferation blockage, apoptosis, injury and other detrimental effects (Jomova et al., 2023; Liguori et al., 2018). Cancer cells typically possess higher levels of ROS than normal cells and are more sensitive to elevated ROS levels, implying different levels of oxidative stress under certain conditions determine the fate of tumor cells (Guo et al., 2020). Compelling evidences have demonstrated that accumulated ROS levels activate the ER stress response (Cui et al., 2022; Singh-Mallah et al., 2019). Sea cucumber extracts have been shown to induce ROS production, hence stimulating c-Jun N-terminal kinase (JNK) and ER stress-related apoptotic pathways in CRC (Kim et al., 2019). In addition, p20BAP31 promoted calcium (Ca^2+^) release and raised ROS generation, leading to ER stress in CRC (Jiang et al., 2023). Nicotinamide adenine dinucleotide phosphate (NADPH) oxidases (NOXs) are key enzymes involved in the generation of ROS, playing a crucial role in the redox signaling. Aberrant NOXs expression may lead to the ROS-mediated apoptosis (Block and Gorin, 2012). It has been reported that anlotinib induces ROS production and suppresses mitochondrial respiration by NOX5-mediated redox imbalance (Huang et al., 2021). Thus, selective modulation of ROS represents a powerful strategy for anti-cancer therapy, and targeting NOXs-ROS pathway holds great potential for effective anti-cancer drug development.

In this study, we confirmed the efficacy of combining PD with OXA in CRC treatment. PD exerted anti-tumor activity by stimulating ER stress and DNA damage via the NOX5-ROS pathway. In addition, the combination of PD and OXA had a synergistic effect, resulting in significant accumulation in ROS-mediated ER stress and DNA damage. The combination of OXA and PD is a promising new treatment for CRC, and targeting NOX5 may offer new avenues for CRC therapy.

## 2 Materials and methods

### 2.1 Cell culture

Human CRC cell lines RKO (CVCL number: 0504; catalogue number: TCHu116; Shanghai, China) and LoVo (CVCL number: 0399; catalogue number: SCSP-514; Shanghai, China) were provided from the Institute of Biochemistry and Cell Biology, Chinese Academy of Sciences. The cells were respectively cultured in F12K and RPMI 1640 medium (Procell Life Science & Technology, Wuhan, China) added with 10% fetal bovine serum (FBS; YAMAY Biotech, Shanghai, China) and 1% penicillin-streptomycin (New Cell & Molecular Biotech, Suzhou, China), and cells were digested using 0.25% Trypsin-EDTA (New Cell & Molecular Biotech, Suzhou, China). Normal human liver cells (MIHA) were purchased from Cell Bank of Chinese Academy of Sciences (Shanghai, China), and normal colon epithelial cells (FHC) were obtained from BioVector NTCC Inc. (Beijing, China). The cells were cultured in RPMI 1640 medium with 15% or 10% FBS respectively. All cells were incubated at 37°C in a humidified incubator with 5% CO_2_ atmosphere.

### 2.2 Reagents and antibodies

PD was obtained from Selleck Chemicals (Cat# 27208-80-6, purity 99%). OXA was supplied from MedChemExpress (Cat# HY-17371/CS-0992). 3-(4,5-dimethylthiazol-2-yl)-2,5-diphenyltetrazolium bromide (MTT, Cat# M8180) was obtained from Solarbio Science & Technology (Beijing, China). ROS probe 2′, 7′ -dichlorodihydrofluorescin diacetate (DCFH-DA) was acquired by Beyotime (Shanghai, China). N-acetyl-L-cysteine (NAC, Cat# A7250) was purchased from Sigma-Aldrich (St. Louis, MO, United States). 4-phenylbutyric acid (4-PBA, Cat# 151280) was supplied from TargetMol (Boston, MA, United States). TB Green Premix Ex Taq (Tli RNase H Plus, Cat# RR820A) was purchased from Takara Bio Inc. Antibodies against phospho-H2AX-S139 (Cat# AP0687, RRID: AB_2863808, 1:2000) was provided from ABclonal (Wuhan, China). Glyceraldehyde-3-phosphate dehydrogenase (GAPDH, Cat# 2251-1, RRID: AB_1267174, 1:10000) was provided from Abcam (Waltham, MA). HRP-linked anti-rabbit IgG antibody (Cat# 7074S, 1:2000) and HRP-linked anti-mouse IgG antibody (Cat# 7076S, 1:2000) were purchased from Cell Signaling Technology (Danvers, MA, United States). CHOP (Cat# 66741-1-Ig, RRID: AB_2882089, 1:1000), activating transcription factor 4 (ATF4, Cat# FITC-10835, RRID: AB_2883737, 1:1000), Vinculin (Cat# CL594-26520, RRID: AB_2919877, 1:5000) and NOX5 (Cat# 25,350-1-AP, RRID: AB_2811208, 1:1000) were supplied from Proteintech (Wuhan, China).

### 2.3 Cell viability assay

The LoVo and RKO cells were resuspended and inoculated into 96-well plates at a density of 5.5 × 10^3^ or 5 × 10^3^ per well respectively. Additionally, FHC and MIHA cells were seeded at a density of 3 × 10^3^/well and incubated for 24 h. PD was used within the following range of concentrations (0, 100, 200, 300, 400, 500, 600 μM). At the end of the incubation period, 25 µL of MTT reagent was mixed into each well and incubated for an additional 3 h. The absorbance was measured using a SpectraMax iD3 instrument (Molecular Devices, LLC), and the cell growth curves were plotted based on the corresponding normalized values. For the combination treatment, the LoVo and RKO cells were seeded into 6-well plates at a density of 2–3 × 10^5^ per well, and exposed to 400 μM PD and various concentrations of OXA (20, 40, 60, 80, 100 μM) for 48 h. In addition, LoVo and RKO cells were treated with 400 μM PD following NAC (5 mM) or 4-PBA (0.4 mM) pretreatment. Cell viability was assessed by counting the cell numbers following trypan blue staining. Combination index (CI) was calculated by using CompuSyn 2.0 software (http://www.Combosyn.com/index.html) to assess drug-drug interactions. A CI value of 1 indicates additive interaction, whereas CI >1 or CI <1 indicates antagonistic or synergistic interactions, respectively.

### 2.4 Colony formation assay

LoVo and RKO cells were cultured in six-well plates at a density of 1,000 cells per well for 24 h. The different concentrations of PD were added into RKO (100, 200, 300, 400 μM) and LoVo (100, 150, 200, 300 μM) cells. For the combination treatment, the cells were treated with PD (200 μM), OXA (1 μM) or their combination with or without 5 mM NAC pretreatment for 1 h. After 7–14 days, 4% paraformaldehyde (PFA) was used to fix cells for 15 min. Then the cells were stained with 0.5% crystal violet and colonies were counted by ImageJ (version 1.53t, U. S. National Institutes of Health).

### 2.5 Wound-healing assay

The cells (3 × 10^5^) were plated in a 6-well plate and incubated at 37°C for 24 h to form monolayer. Then using a sterile 10 μL pipette tip to make scratches in the cell monolayer. Wash the cells with 1 × PBS to remove floating cells and replaced with fresh media supplemented with 2% FBS. The wounded area was imaged immediately using an inverted microscope. Then the cells were further treated with PD (200 μM), OXA (1 μM), or combination thereof with or without 5 mM NAC pretreatment for 1 h. After 72 h of incubation, wounded area was photographed and analyzed by ImageJ software (version 1.53t, U. S. National Institutes of Health).

### 2.6 ROS assay

The cells were inoculated into 6-well plates and incubated at 37°C for 24 h, and treated with 400 μM PD for different times (RKO: 3, 6, 9, 12 h; LoVo: 1, 2, 4, 6, 8 h) as experiments needed. For combination group, the cells were exposed with 40 μM of OXA, 400 μM of PD, or their combination with or without NAC pretreatment. After a certain time of action, cells were stained with 10 μM DCFH-DA and maintained at 37°C for 30 min with light protection. ROS production (DCF fluorescence) was measured using a FACS Calibur flow cytometer (BD Biosciences, CA) and the average fluorescence intensity was analyzed using FlowJo software (Tree Star, Inc.). The number of 10,000 events were counted for each sample.

### 2.7 Western blot

Treated cells were harvested, washed once with PBS, and total proteins were extracted with RIPA lysis buffer (Cat# AR0103-100, Boster bio). Protein assay kit (Bio-Rad, Herculer, CA) was used to measure protein concentration. Equal amounts of total protein were separated by electrophoresis on sodium dodecyl sulfate polyacrylamide gel electrophoresis (SDS-PAGE) gels and then transferred to nitrocellulose membranes. After being blocked with 5% skimmed milk powder solution for 1.5 h, the membrane was cleaned three times using tris buffered saline with Tween 20 (TBST) and then incubated overnight with specific primary antibodies at 4°C with gentle shaking. The membrane was then washed three times with TBST and reacted with HRP-conjugated anti-mouse IgG antibody for 1 h at room temperature. Densitometric analysis was performed by enhanced chemiluminescence (ECL) exposure imaging using ImageJ software (version 1.53t, NIH).

### 2.8 Transient transfection of small interfering RNA (siRNA)

Cells were inoculated into 6-well plates with 2 × 10^5^ cells per well to achieve 50% cell confluence at the time of transfection. Negative control siRNA and siRNA against NOX5 were transfected into LoVo and RKO cells using lipofectamine 2000 (Invitrogen, Carlsbad, CA, United States) in accordance with the manufacturer’s (Genepharma Inc., Shanghai, China) protocol. After a certain time of transfection, the transfected cells were tested by RT-qPCR and Western blot. The sequences of siRNA are shown in Supplementary Table S1.

### 2.9 Immunofluorescence for γ-H2AX

Immunofluorescence for γ-H2AX was conducted as described previously (Chen et al., 2024). To detect DNA damage, cells were inoculated in 6-well plates and treated with PD (400 µM), OXA (40 µM), or their combination with or without NAC pretreatment. The cells were incubated with anti-phospho-H2AX (Ser139) for 1 h. After washing, the cells were further incubated with CoraLite 488-conjugated Goat Anti-Rabbit IgG (H + L) antibodies. After 4′,6-diamidino-2-phenylindole (DAPI) staining, fluorescence intensity was evaluated by using orthogonal microscopy (Laica, Germany).

### 2.10 Real-time quantitative polymerase chain reaction (RT-qPCR)

The cells were broken by TRIzol (Invitrogen, Carlsbad, CA, United States), and RNA was isolated with trichloromethane and isopropyl alcohol (Hongsheng Fine Chemical Co., Changshu, China), then RNA (1 μg) was reverse transcribed into cDNA with PrimeScript RT Master Mix (order no. RR036A; Takara Biotechnology Co.), TB Green Premix Ex Taq II reagent (Art. No. RR820A; Takara) was added for RT-qPCR as per the manufacturer’s instructions. All data was repeated three times, and the relative gene expression was detected by the 2^−ΔΔCq^ method (Livak and Schmittgen, 2001). The designated primer sequences used for gene amplification are shown in Supplementary Table S2.

### 2.11 Molecular docking

AutoDock Vina (version 1.5.6) was conducted to interconnect PD with NOX5 to assess the binding affinity. The structure pdb file of NOX5 (8U86) was obtained from PDB (https://www.rcsb.org/). PD structure (CID: 73642) was extracted from the PubChem (https://pubchem.ncbi.nlm.nih.gov/). The docking grid dimensions in the docking process were set to 78, 86, and 78 Å, while the center coordinates for X, Y, and Z were specified as 133.037, 132.480, and 142.930, respectively. The PyMOL Molecular Graphics System (Version 2.6.0) was used for the visualizations and graphics generations of PD and NOX5.

### 2.12 Statistical analysis

Results were presented with means ± standard deviation (SD) of three independent experiments (n = 3). Data from multiple groups were analyzed using one-way ANOVA with Tukey’s multiple comparisons test. All statistical analyses were performed using GraphPad Prism 9.0.2 (GraphPad Software, San Diego, CA, United States). A *P*-value of <0.05 has a significant difference.

## 3 Results

### 3.1 PD inhibited colon cancer cell growth by inducing ROS accumulation and ER stress

**FIGURE 1:**
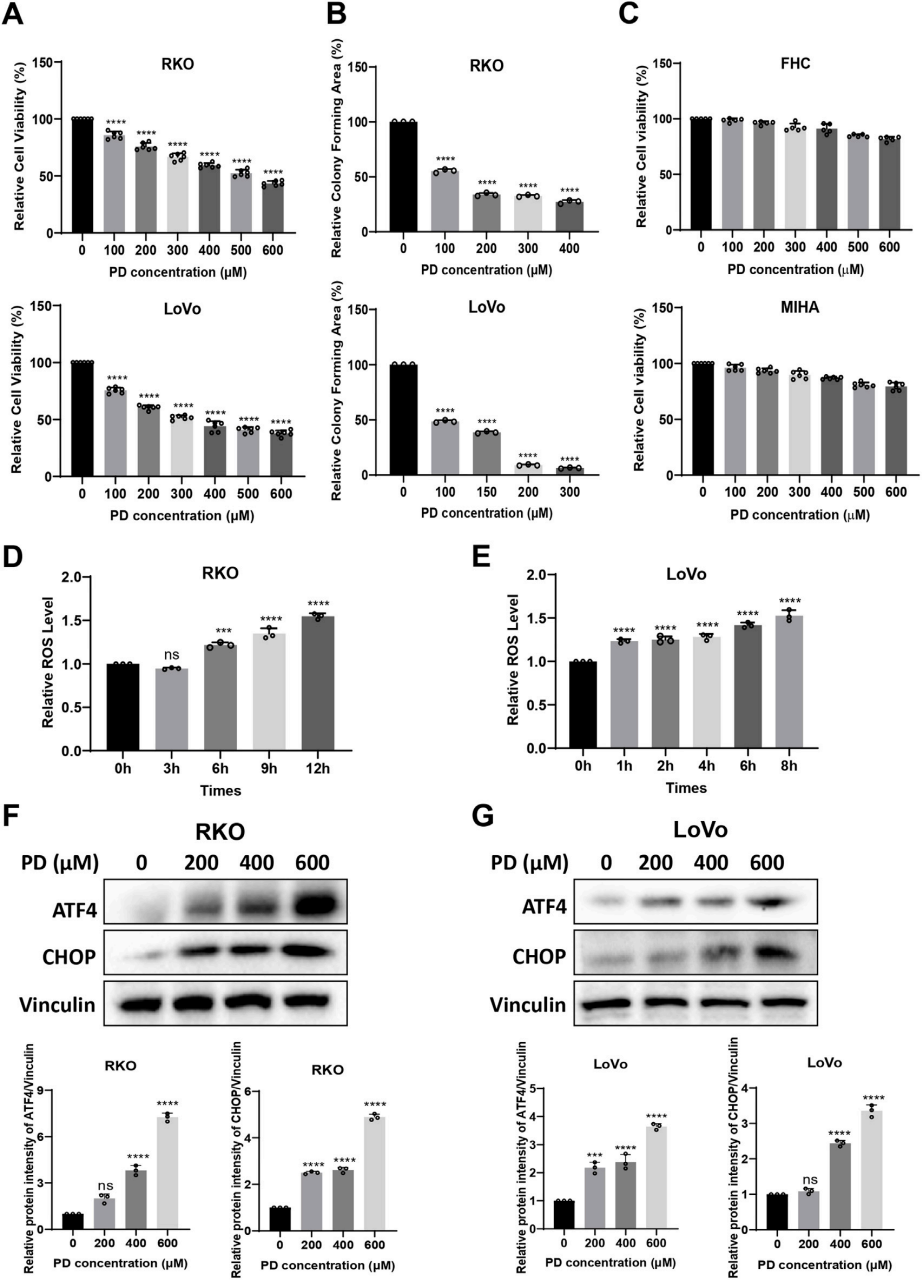
PD exerted anti-tumor activity by inducing ROS generation and ER stress. **(A–C)** RKO, LoVo, FHC and MIHA cells were treated with the increasing concentrations of PD, cell viability was assayed by MTT assay **(A, C)** and colony forming ability was assayed by colony formation assay **(B)**. **(D, E)** The intracellular ROS level was detected using DCFH-DA (10 μM) probe after treatment with PD (400 μM) at different time points in RKO **(D)** and LoVo **(E)** cells. **(F, G)** Western blot detects the expression of ATF4 and CHOP after treatment with different concentrations of PD in RKO **(F)** and LoVo **(G)** cells. The band intensity was quantified by ImageJ software. Values represent means ± SD (n = 3). **P < 0.01, ***P < 0.001, ****P < 0.0001.

Increasing evidence suggests that PD has anti-tumor effects in a variety of cancers (Moloney et al., 2017). To better understand the effects of PDs in colon cancer cell growth, the cytotoxicity of PD in RKO and LoVo (Figure 1A) cells was examined by MTT assay. PD was shown to inhibit the growth of colon cancer cells in a concentration dependent manner. In addition, the number of colonies formed by RKO and LoVo (Figure 1B; Supplementary Figures S1A, B) cells were significantly decreased with increasing PD concentration, indicating that PD exerts anti-CRC activities. Furthermore, we investigated the cytotoxicity of PD in normal cells, FHC and MIHA. PD exhibited minor cytotoxicity against FHC and MIHA cells, with 600 μM PD inhibiting both FHC and MIHA cell growth by only about 15%–20% compared to the non-treatment group (Figure 1C).

Cancer cells are more sensitive to increase in intracellular ROS levels (An et al., 2024; Verma and Tiku, 2022). Elevated ROS levels promote anti-cancer signaling and initiate oxidative stress-induced cancer cell death (Perillo et al., 2020). In order to further investigate the mechanism of action of PD on colon cancer cells, we measured the ROS levels after PD treatment. The ROS levels in RKO and LoVo cells were elevated in a time-dependent manner (Figures 1D, E). These results suggested that PD inhibits colon cancer cell growth by promoting the ROS accumulation.

Prolonged accumulation of misfolded or unfolded proteins increases intracellular ROS levels, inducing ER stress and triggering the unfolded protein response (UPR), which includes activation of the ATF4-CHOP pathway (Liu et al., 2006). Therefore, we examined the expression of ER stress-related proteins in colon cancer cells after treatment with PD. As shown in Figures 1F, G, the expression levels of ATF4 and CHOP proteins were markedly increased in a concentration-dependent manner following PD treatment in RKO and LoVo cells. All these results suggested that PD may induce cell death by elevating ROS production and activating ATF4 and CHOP signaling pathway in colon cancer cells.

### 3.2 ROS inhibitor attenuated PD-induced colon cancer cell death and ER stress

**FIGURE 2:**
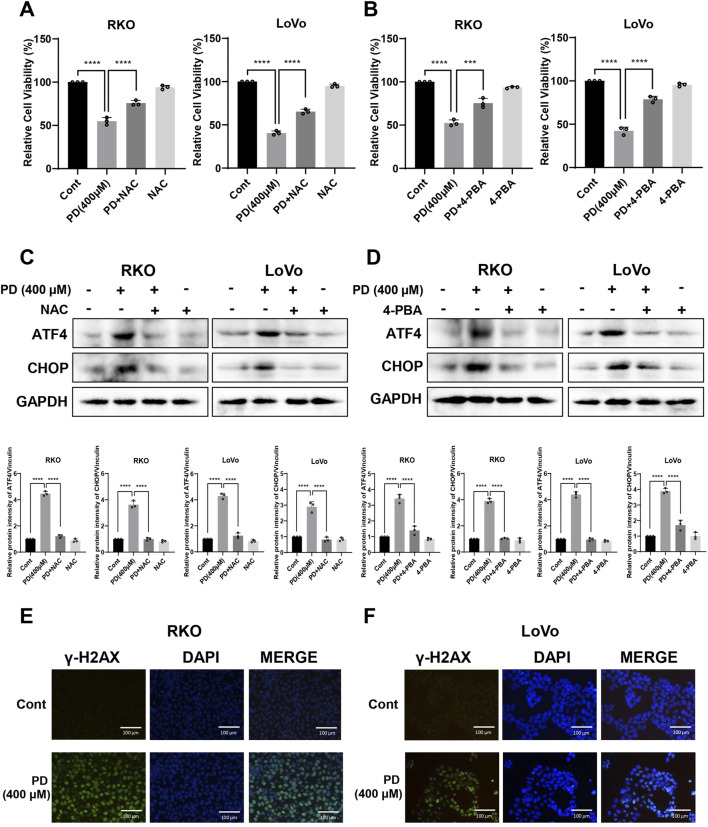
PD treatment triggered ROS-mediated ER stress and DNA damage. **(A–D)** Colon cancer cells were treated with PD (400 μM) followed by 5 mM NAC **(A)** or 0.5 mM 4-PBA **(B)** pretreatment. Cell viability was evaluated by counting cell numbers after trypan blue staining, and the expressions of ATF4 and CHOP **(C, D)** were detected by Western blot analysis. **(E, F)** Representative images of γ-H2AX immunofluorescence in RKO **(E)** and LoVo **(F)** cells following PD treatment. Green immunofluorescence shows expression of γ-H2AX, blue immunofluorescence shows DAPI staining, scale bar = 200 μm. Values represent means ± SD (n = 3). ***P < 0.001, ****P < 0.0001.

To demonstrate the critical role of ROS in PD-mediated colon cancer cell death and ER stress, we treated cells with the ROS scavenger NAC. As expected, pretreatment with NAC significantly reduced inhibitory effects of PD (Figure 2A; Supplementary Figure S1C) and notably reversed the protein expressions of ATF4 and CHOP in colon cancer cells (Figure 2C). Similarly, pretreatment of colon cancer cell with an ER stress inhibitor, 4-phenylbutyric acid (4-PBA), markedly attenuated PD induced cell death (Figure 2B; Supplementary Figure S1D) and the expressions of ATF4 and CHOP (Figure 2D), suggesting that ROS-mediated ER stress plays a key role in the PD-mediated cell death.

Excessive ROS accumulation induces DNA damage (Srinivas et al., 2019). To evaluate the effects of PD on DNA damage in colon cancer cells, the expression level of γ-H2AX was detected using a cellular immunofluorescence assay. The results showed that PD treatment increased the fluorescence level of γ-H2AX in both RKO (Figure 2E) and LoVo (Figure 2F) cells, suggesting that PD induces DNA damage in colon cancer cells.

### 3.3 PD combined with OXA enhanced the cytotoxicity against colon cancer cells

**FIGURE 3:**
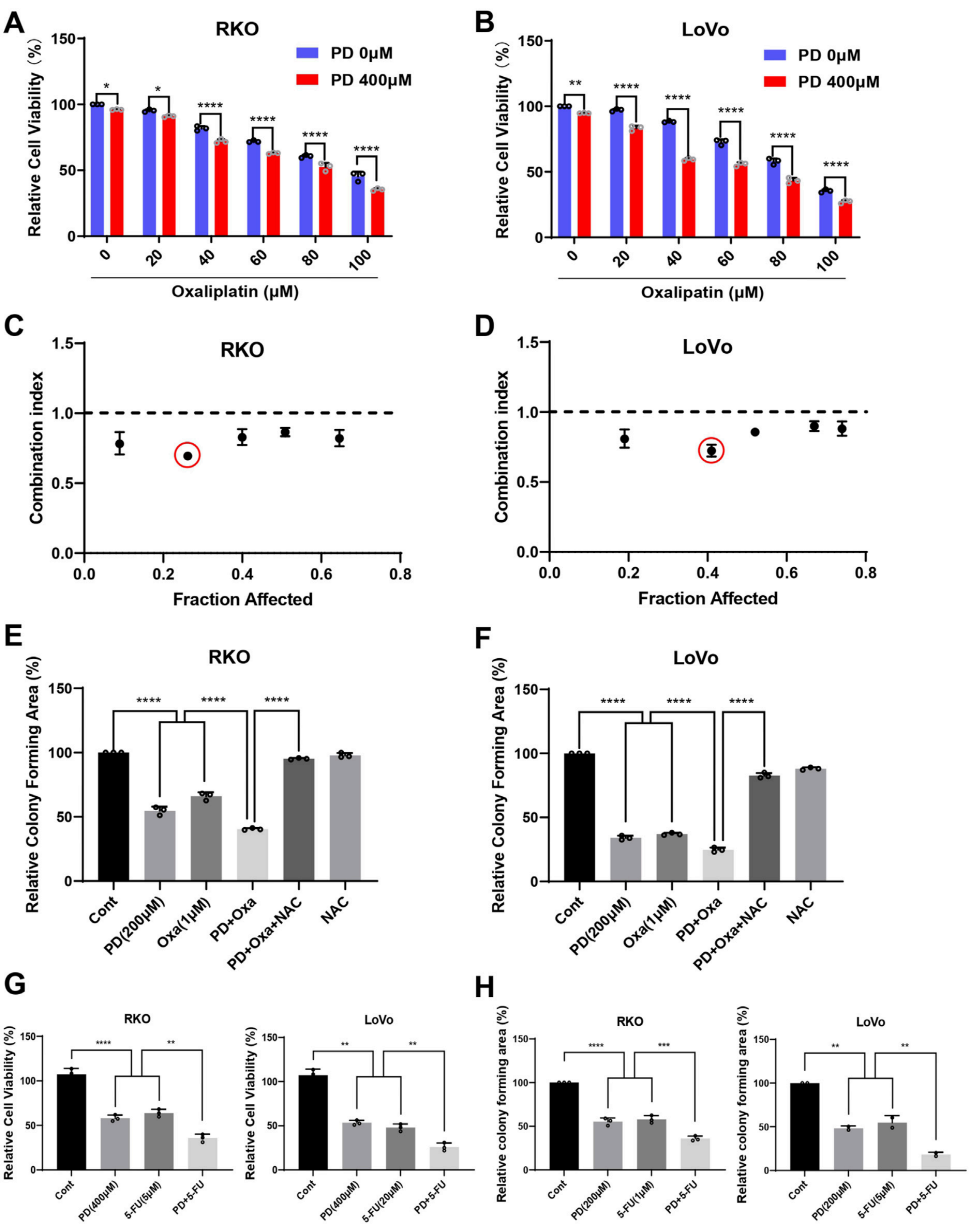
PD combined with OXA enhances the cytotoxicity in colon cancer cells. **(A, B)** Cell viability was measured after treatment with PD (400 μM) and OXA (0, 20, 40, 60, 80, 100 μM) for 24 h in RKO **(A)** and LoVo **(B)** cells. **(C, D)** The CI values were calculated from the percentage of growth inhibition using CompuSyn software. CI < 1.0 is considered as synergistic. **(E, F)** Colony formation assay was conducted following treatment with PD (200 μM), OXA (1 μM) or their combination with or without NAC pretreatment in RKO **(E)** and LoVo **(F)** cells. **(G)** The cells were treated with PD (400 μM), 5-FU (5 μM for RKO and 20 μM for LoVo cells), or their combination, and the cell viability was measured by counting cell numbers after trypan blue staining. **(H)** Colony forming ability was evaluated following treatment with PD (200 μM), 5-FU (1 μM for RKO and 5 μM for LoVo cells), or their combination. ImageJ software was used to evaluate colony forming area. Results are summarized as mean ± SD (n = 3). *P < 0.05, **P < 0.01 and ***P < 0.001.

As is widely acknowledged, OXA induces ROS, which contributes to its anti-tumor activity (Al-Otaibi and Almotwaa, 2022; Kwak et al., 2023). To investigate whether the combination treatment of OXA and PD exerts synergistic anti-tumor activities, we evaluated the proliferative ability of RKO and LoVo cells following single-agent or combination treatments. The combination group inhibited colon cancer cell growth in a dose-dependent manner. Additionally, 400 μM of PD significantly increased the cytotoxicity of OXA in both RKO (Figure 3A) and LoVo cells (Figure 3B). The interaction between PD and OXA was assessed by calculating the drug combination index (CI) value using CompuSyn software. Among the different combination groups, the combination of 40 μM OXA and 400 μM PD exhibited the most pronounced synergistic inhibitory effect in both RKO (Figure 3C) and LoVo (Figure 3D) cells, with CI values of 0.69 and 0.72, respectively. These results indicated that PD and OXA exhibit synergistic anti-tumor effects in colon cancer cells.

Next, colony formation assay was conducted to prove whether PD could enhance the inhibitory effect of OXA on colony forming ability in colon cancer cells. The number of colonies formed after the combination treatment with PD and OXA was significantly lower than that in either PD or OXA monotherapy groups (Figures 3E, F; Supplementary Figures S2A, B). Interestingly, NAC, a scavenger of ROS, significantly mitigated the inhibition of colony formation induced by the combination treatment. These data suggest that combination of PD and OXA exerts synergistic anti-CRC effects, which are associated with ROS production. Additionally, PD in combination with 5-FU, another standard first-line treatment option for CRC, significantly inhibited colon cancer cell viability (Figure 3G; Supplementary Figures S3A, B) and colony formation (Figure 3H; Supplementary Figures S3C, D) compared to the single treatment alone.

### 3.4 The combination of PD and OXA enhanced DNA damage by increasing ROS levels

**FIGURE 4:**
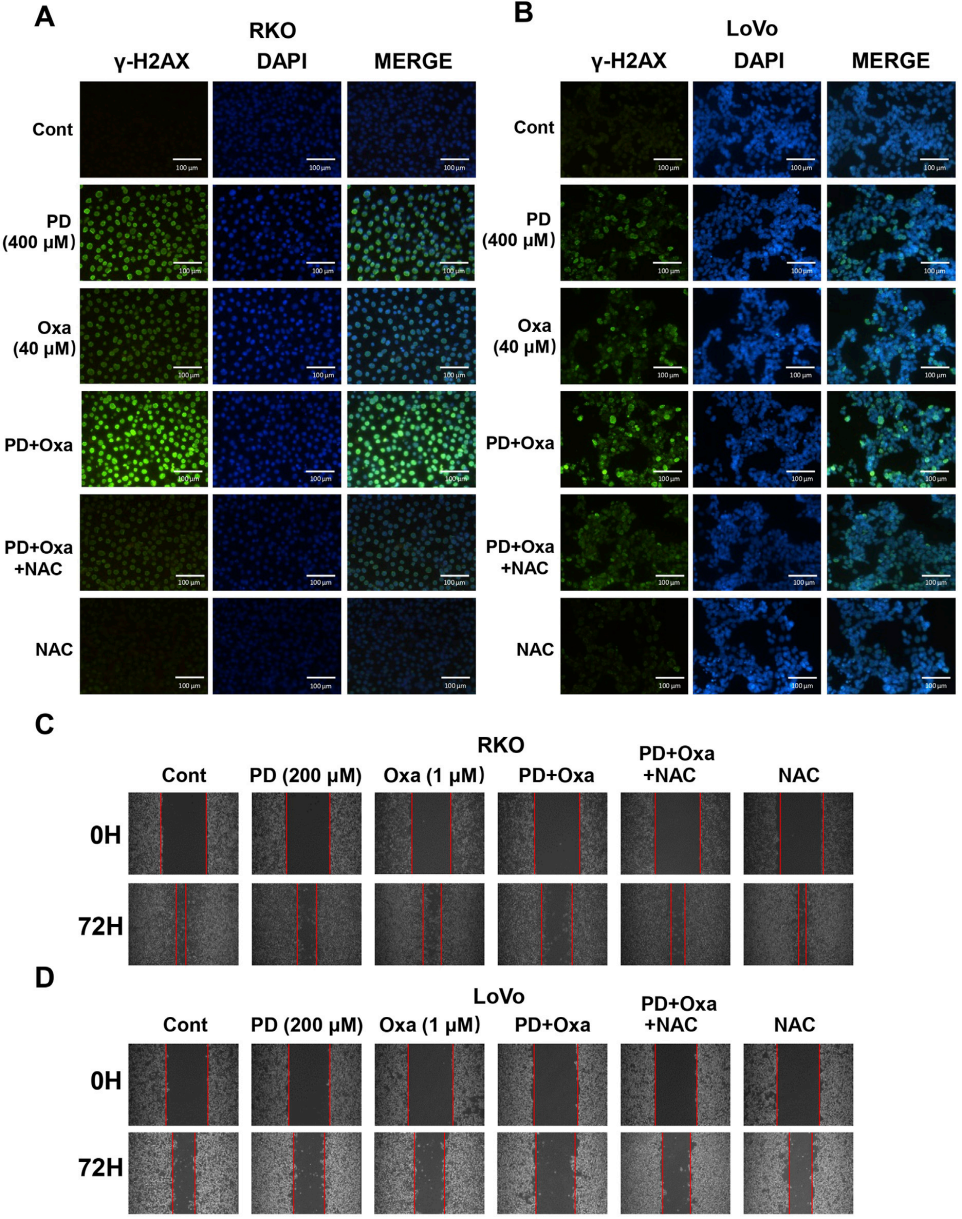
PD in combination with OXA enhanced DNA damage in colon cancer cells. **(A, B)** Immunofluorescence detection of γ-H2AX in RKO **(A)** and LoVo **(B)** cells following treatment with PD (400 μM), OXA (40 μM) or combination theirof. Scale bar = 200 μm. **(C, D)** Cell migratory ability was evaluated by wound-healing assay after 200 μM PD, 1 μM OXA, or their combination with NAC pretreatment in RKO **(C)** and LoVo **(D)** cells.

OXA exerts cytotoxicity by inducing DNA damage (Riddell, 2018). Thus, we conducted cellular immunofluorescence assays to determine whether PD enhances DNA damage caused by OXA in colon cancer cells. The combination of PD (400 μM) and OXA (40 μM) produced a stronger fluorescence intensity of γ-H2AX than that of the single-drug treatment groups in both RKO (Figure 4A) and LoVo (Figure 4B) cells, indicating that PD combined with OXA exerts anti-CRC activity by inducing DNA damage. The wound healing assay also demonstrated that the combination treatment markedly restricted the migration in colon cancer cells (Figures 4C, D). As expected, NAC pre-treatment effectively reversed these effects.

### 3.5 Combined treatment with PD and OXA stimulated ROS-mediated ER stress

**FIGURE 5:**
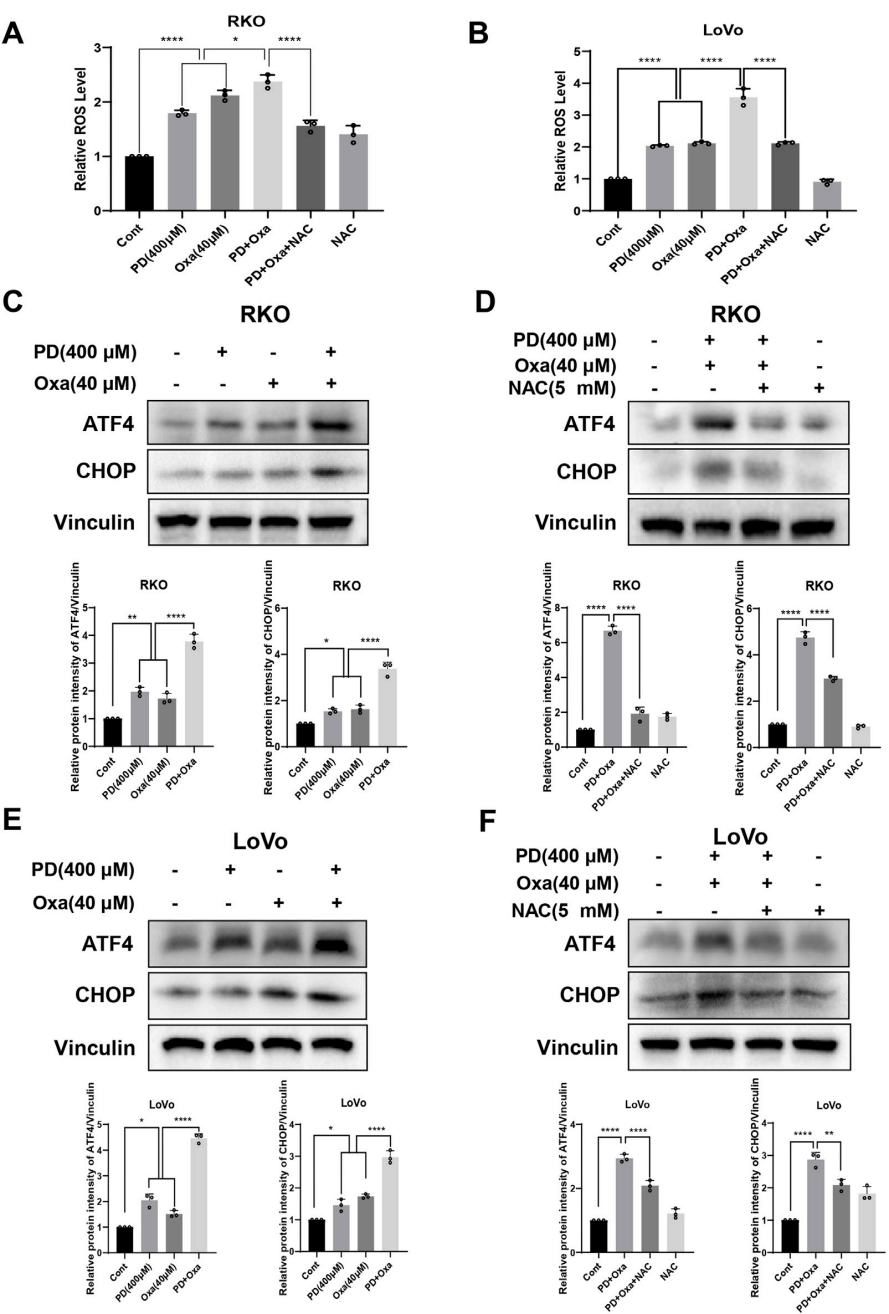
PD combination with OXA induces ROS-mediated ER stress. **(A, B)** The ROS levels were measured after treatment with PD (400 μM), OXA (40 μM) or their combination following NAC (5 mM) pretreatment for 1 h by flow cytometry in the RKO **(A)** and LoVo **(B)** cells (n = 3). **(C–F)** The protein levels of ATF4 and CHOP were detected after treatment with PD, OXA or their combination with or without pretreatment with NAC by Western blot in both RKO **(C, D)** and LoVo **(E, F)** cells. Vinculin was used as internal control. Band intensity was calculated using ImageJ software, and all results were expressed as mean ± SD (n = 3). **p < 0.01 and ***p < 0.001, ****p < 0.0001.

A previous study reported that OXA induces ROS, which contributes to its anti-tumor activity (Wang et al., 2024). To investigate whether PD enhances ROS levels induced by OXA, we examined ROS levels in colon cancer cells after PD (400 μM), OXA (40 μM), or their combination. As expected, the combination group induced higher ROS levels compared to the PD or OXA single-agent groups in both RKO (Figure 5A) and LoVo (Figure 5B) cells, while the elevated ROS levels were considerably reduced after pretreatment with NAC in the combination group. Expressions of ATF4 and CHOP, key proteins in the ER stress pathways mediated by ROS, were further examined by Western blot analysis in RKO and LoVo cells. Compared with OXA or PD treatment alone, the combination treatment noticeably augmented the expression levels of ATF4 and CHOP in both RKO (Figure 5C) and LoVo cells (Figure 5E), and this effect was reversed by NAC pretreatment (Figures 5D, F). Collectively, these results suggest that combination of PD and OXA stimulates ROS-mediated ER stress pathway in colon cancer cells.

### 3.6 PD activates ROS-mediated ER stress pathway by promoting NOX5 expression

**FIGURE 6:**
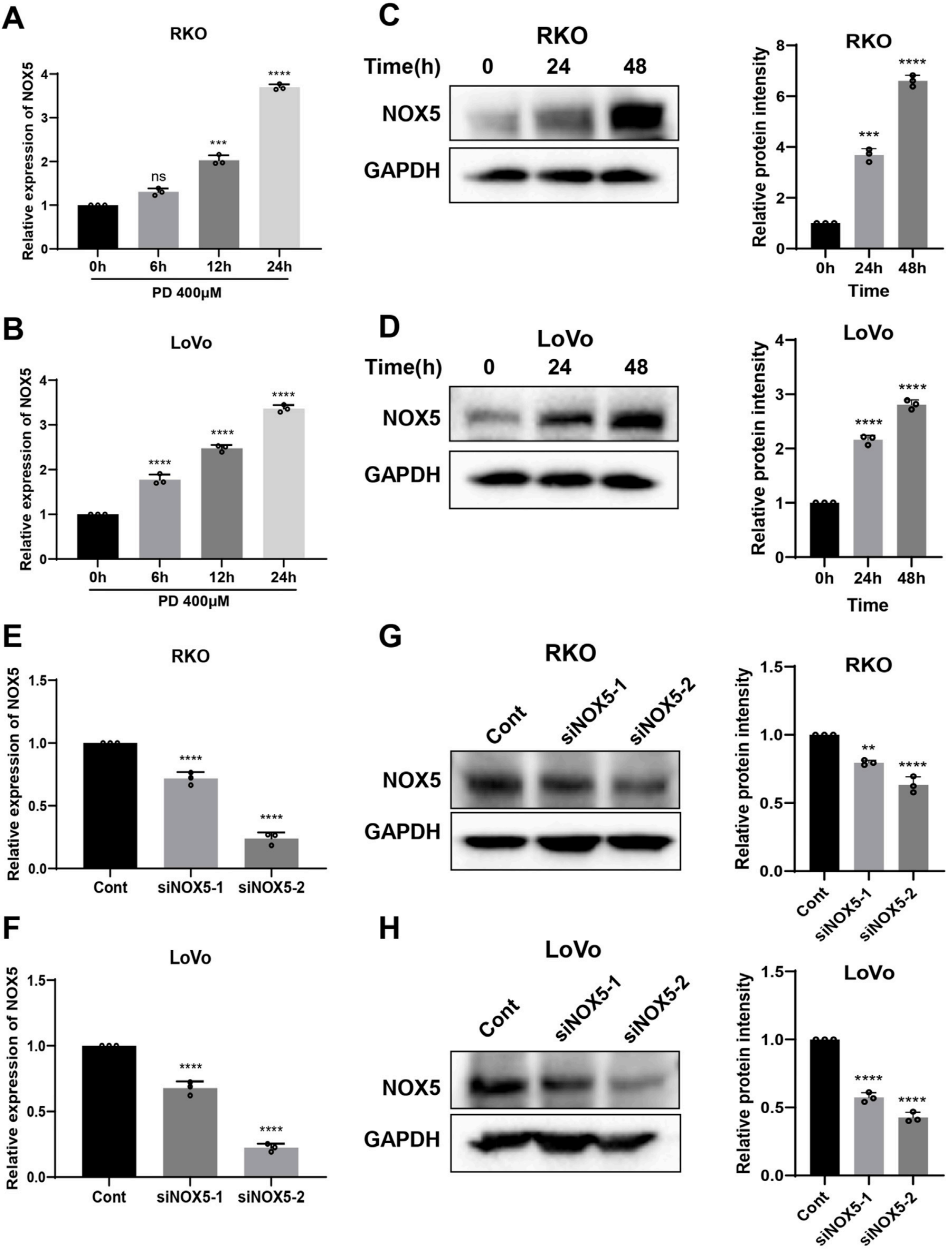
PD treatment increased NOX5 expression in colon cancer cells. **(A, B)** The RT-qPCR assay was used to detect the mRNA expression of NOX5 after treatment with PD at different time points in RKO **(A)** and LoVo **(B)** cells. **(C, D)** Western blot assay was used to detect the levels of NOX5 protein following treatment with PD at different times in RKO **(C)** and LoVo **(D)** cells. GAPDH was used as an internal control. **(E, F)** The NOX5 mRNA expression was detected by qRT-PCR in RKO **(E)** and LoVo **(F)** cells after silencing NOX5. **(G, H)** Western blot was performed to detect NOX5 protein levels in the RKO **(G)** and LoVo **(H)** cells after silencing NOX5. Band intensity was calculated using ImageJ software, and the results were expressed as mean ± SD (n = 3). **P < 0.01, ***P < 0.001, ****P < 0.0001.

**FIGURE 7:**
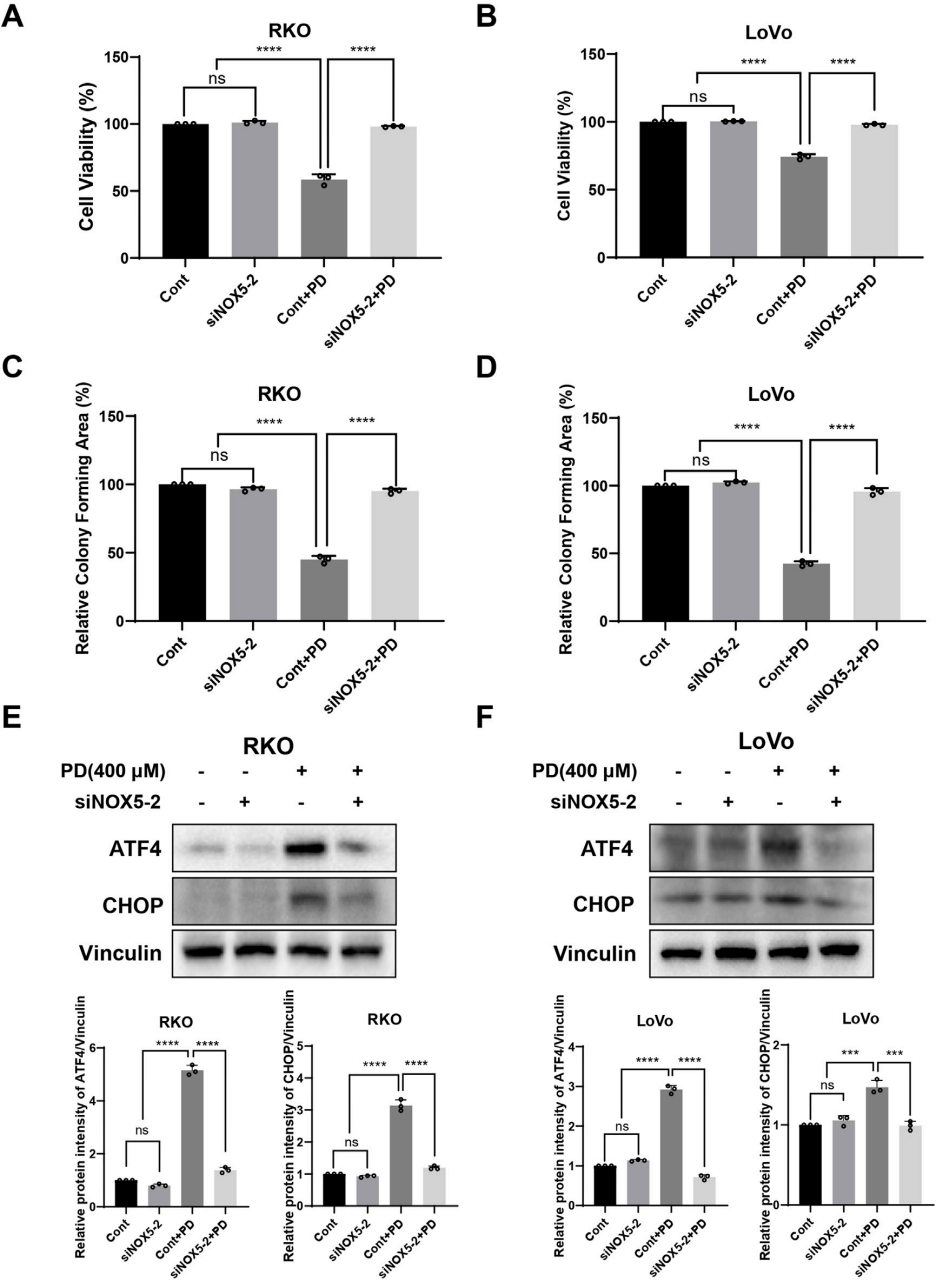
NOX5 knockdown attenuated PD induced ER stress pathway in colon cancer cells. **(A–D)** NOX5 knockdown or control colon cancer cells were treated with PD. Cell viability was evaluated by counting cell numbers after trypan blue staining **(A, B)**, and colony formation assays **(C, D)** were conducted to evaluate the colony forming ability. **(E, F)** NOX5 knockdown or control cells were treated with PD, and the ATF4 and CHOP expression was detected by Western blot analysis. Vinculin was used as internal control. Band intensity was calculated using ImageJ software, and results are summarized as mean ± SD (n = 3). **P < 0.01, ***P < 0.001, ****P < 0.0001.

The NOXs complex, consisting of seven isoforms, is responsible for driving cellular ROS production in response to extracellular stimuli, including NOX4 and NOX5 (Bedard et al., 2012; Doroshow and Kummar, 2014). Therefore, we investigated NOX4 and NOX5 expression levels following PD treatment. The PD treatment significantly elevated both mRNA and protein levels of NOX5 in colon cancer cells (Figures 6A–D), while did not significantly change the expression of NOX4 mRNA (Supplementary Figure S4A). The molecular docking result further predicted that the lowest binding affinity between PD and NOX5 was −8.1 kcal/mol, verifying top-ranked docking pose between PD and NOX5 (Supplementary Figure S4B). PD formed two carbon-hydrogen bonds with conventional amino acid residue THR-248 and TYR-525, two hydrogen bonds with amino acid residue ARG-245 and ASP-522 respectively in NOX5 activate pocket, suggesting that potential significant interactions between NOX5 and PD. To further determine the effect of NOX5 in colon cancer cells, we knocking down NOX5 expression by transfecting siRNAs. After transfection with siRNAs (siNOX5-1 and siNOX5-2) into colon cancer cells, NOX5 mRNA and protein levels were considerably downregulated in both RKO and LoVo cells (Figures 6E–H). Notably, the silencing effect of siNOX5-2 was stronger than that of the siNOX5-1, thus we used siNOX5-2 for further experiments. Knockdown of NOX5 did not affect the proliferative and colony forming ability of colon cancer cells, but it attenuated the inhibitory effect of PD on cell viability (Figures 7A, B; Supplementary Figures S5A, B) and cell colony formation (Figures 7C, D; Supplementary Figures S5C, D). Furthermore, silencing NOX5 reversed the elevation of ATF4 and CHOP following PD treatment (Figures 7E, F), suggesting that NOX5 is a key mediator in the PD-induced ROS-mediated ER stress pathway in colon cancer cells.

## 4 Discussion

**FIGURE 8:**
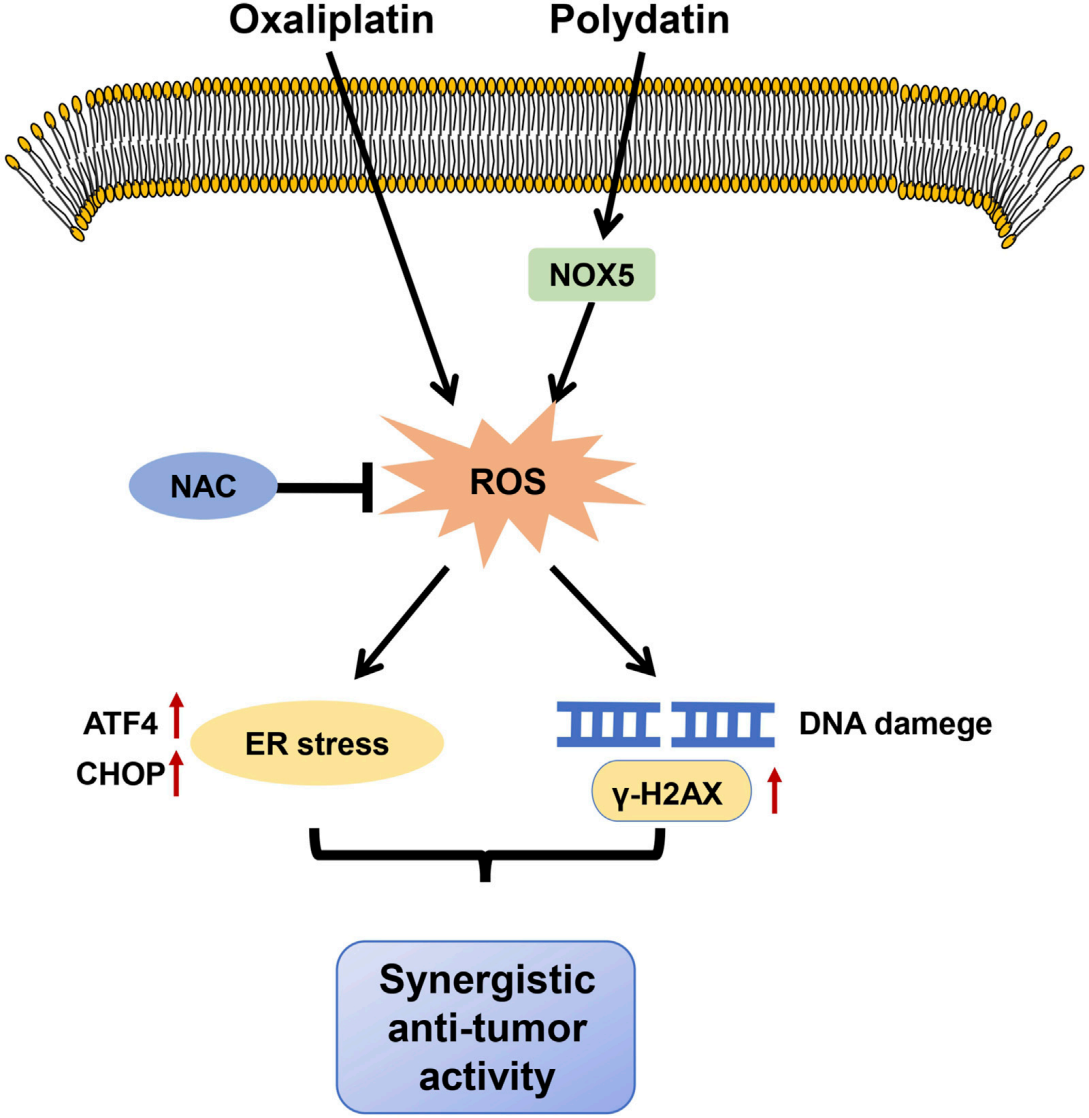
The proposed mechanism of our main findings. PD potentiated anti-tumor activities of OXA by stimulating ROS-mediated ER stress through increasing NOX5 expression and triggering DNA damage.

Increasing evidences suggest that drug combinations provide better therapeutic efficacy than single agents and can significantly reduce the adverse effects of treatment (Martinez-Balibrea et al., 2015). In general, natural products have relatively low cytotoxicity. Therefore, anti-cancer drugs derived from natural sources hold great promise as chemo-sensitizers for cancer treatment (Banerjee et al., 2009; Fulda and Debatin, 2004). PD has a strong proapoptotic effect in CRC (De Gregorio et al., 2022; De Maria et al., 2013). Nonetheless, the role and mechanism of PD combination with OXA have not been fully investigated in CRC. In this study, we demonstrated that PD treatment markedly induced ER stress and DNA damage by promoting NOX5-mediated ROS generation, thereby inhibiting the growth of colon cancer cells. Combining PD with OXA synergistically exerted anti-CRC activity by inducing ROS-mediated ER stress and DNA damage (Figure 8).

DNA damage response (DDR) pathway is crucial for repairing DNA damage and stabilizing the normal cellular genome (Nuciforo et al., 2007). DDR can be used as a therapeutic target for tumor treatment and is closely associated with chemotherapy outcomes and clinical prognosis (Bartkova et al., 2004). γ-H2AX is involved in DNA damage repair, sustaining genome stability and completeness, and serves as a specific biomarker for characterizing DNA damage (Qu Minmin, 2021; Rogakou et al., 1999). ER stress may suppress DNA damage repair and lead to apoptosis (Yamamori et al., 2013). A previous study reported that PD treatment induces DDR-related proteins such as p53 and p21 and concentration-dependently induces cellular DNA damage in lung cancer cells (Verma and Tiku, 2022). It is widely noted that the anti-tumor activity of OXA is associated with the induction of cellular DNA damage (Hochster and Sargent, 2016; Malla et al., 2021). However, few research has specifically focused on the mechanism of cellular DNA damage stimulated by the combination of PD and OXA in CRC. In this research, we investigated DNA damage after PD, OXA or their combination treatment in colon cancer cells. PD treatment significantly induced DNA damage and strengthened OXA triggering DNA damage in colon cancer cells. Additionally, the ROS scavenger reversed the DNA damage caused by the combination treatment with PD and OXA, suggesting that PD enhances OXA-mediated cell death through ROS-mediated DNA damage.

Triggering UPR-mediated ER stress in cancer cells contributes to cell death, of which CHOP and ATF4 act as the downstream gene of UPR sensors (Wu et al., 2020). It has been reported that thioridazine induces immunogenic cell death by activating eIF2α/ATF4/CHOP axis along with secretory autophagy in CRC cells (Tran et al., 2023). Combination treatment with withaferin-A and 5-FU induced apoptosis by increasing the expression of ER stress related proteins CHOP and ATF4 in CRC cells (Alnuqaydan et al., 2020). In our study, we exhibited the role of ROS in PD-induced cell death. We demonstrated that PD treatment induced ROS-mediated ER stress as evidenced by increased ATF4 and CHOP expression. Notably, inhibiting ROS by NAC and blocking ER stress by 4-PBA effectively reversed these effects. Moreover, Combination of PD and OXA exhibited synergistic anti-tumor effects by stimulating ROS-mediated ER stress pathway.

NOX5 protein is an essential protein for intracellular ROS generation and are related to physiological and pathological processes, including the signal transduction, stress response, regulation of cell growth and death (Fulton, 2009; Krause, 2004). The NOX5 dysfunction results in transformation and uncontrolled growth of a variety of cancer cells such as prostate cancer, esophageal adenocarcinoma, hairy cell leukemia, pancreatic cancer and esophageal cancer (Brar et al., 2003). Under hypoxic conditions, ROS levels in osteoblasts were significantly reduced by inhibiting the expression of the NOX5, thereby reducing autophagy and apoptosis (He Pengjie et al., 2021). The NOX family is overexpressed in CRC and is related to the prognosis of the patient (Cho et al., 2018). However, the biological functions of NOX5 in CRC remain largely unknown. We found that PD treatment elevated the mRNA and protein levels of NOX5. Knocking down NOX5 attenuated PD-induced ER stress and DNA damage, reversing the anti-CRC activity of PD, suggesting that NOX5 is a vital target of PD and mediates ROS-induced ER-stress and DNA damage in colon cancer cells. These results provide a new preclinical basis for the treatment of CRC with PD.

## 5 Conclusion

We demonstrated that PD synergizes with OXA to activate ER stress by upregulating NOX5 expression and induce DNA damage, thereby inhibiting the growth of colon cancer cells. Therefore, the combination PD and OXA is promising for the treatment of CRC, and targeting NOX5 may offer a new approach for CRC therapy.

## Data Availability

The original contributions presented in the study are included in the article/Supplementary Material, further inquiries can be directed to the corresponding authors.

## Abbreviations

OXA, Oxaliplatin; CRC, colorectal cancer; PD, Polydatin (3,4′,5-trihydroxy-3-β-d-glucopyranoside; NOX5, Nicotinamide Adenine Dinucleotide Phosphate oxidase 5; ER, endoplasmic reticulum; ROS, reactive oxygen species; CHOP, C/EBP-homologous protein; ATF4, activating transcription factor 4; 5-FU, 5-fluorouracil; DHA, Dihydroartemisinin; MAPK, mitogen-activated protein kinase; FBS, fetal bovine serum; DCFH-DA, 2′, 7′ -dichlorodihydrofluorescin diacetate; MTT, 3-(4,5-dimethylthiazol-2-yl)-2,5-diphenyltetrazolium bromide; NAC, N-acetyl-L-cysteine; 4-PBA, 4-phenylbutyric acid; GAPDH, glyceraldehyde-3-phosphate dehydrogenase; ATF4, activating transcription factor 4; SDS-PAGE, sodium dodecyl sulfate polyacrylamide gel electrophoresis; TBST, tris buffered saline with Tween 20; ECL, enhanced chemiluminescence; siRNA, Transient transfection of small interfering RNA; DAPI, 4′,6-diamidino-2-phenylindole; RT-qPCR, Real-time quantitative polymerase chain reaction; UPR, unfolded protein response; DDR, DNA damage response.
