# Supplementary material for: Polydatin enhances oxaliplatin-induced cell death by activating NOX5-ROS-mediated DNA damage and ER stress in colon cancer cells
Source: Front Pharmacol. 2025 Jan 9;15:1532695. doi: 10.3389/fphar.2024.1532695 (PMC11754409; doi:10.3389/fphar.2024.1532695)
Supplement: Supplementary file 1 [file DataSheet1.pdf]

Supplementary information

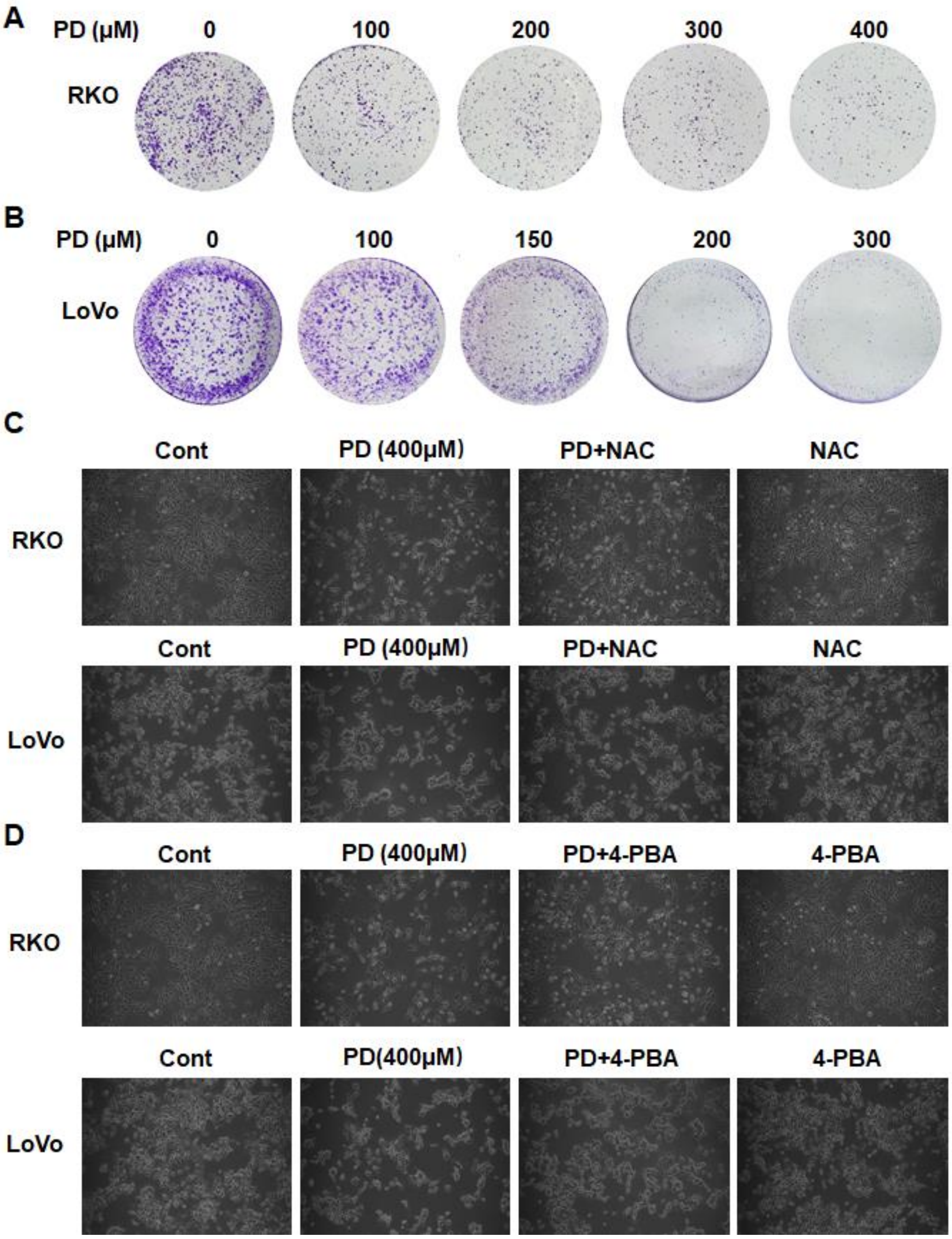

Figure S1. ROS and ER stress inhibitors attenuated anti-tumor effects of PD. (A, B) RKO (A) and LoVo (B) cells were treated with different concentrations of PD, and colony forming ability was detected. Representative pictures were shown. (C, D) Cell viability assays were performed after treatment with PD following NAC (C) or 4-PBA (D) pretreatment. Images were obtained at 100× magnification.

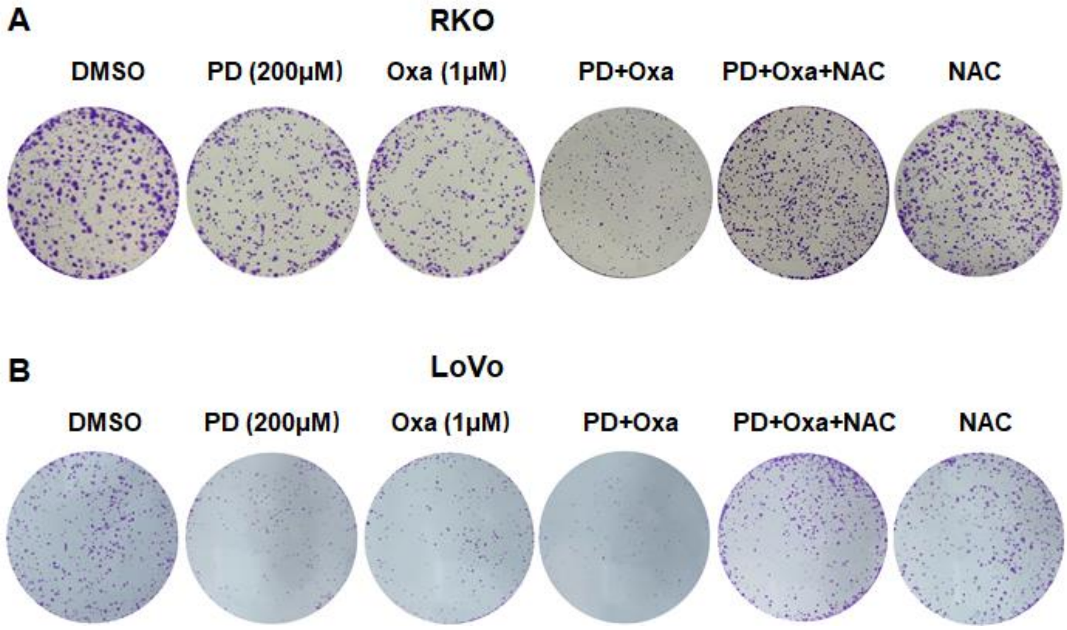

Figure S2. Combination of PD and OXA strongly inhibited colony formation. (A, B) Colony formation assay was performed after treatment with PD (200 μM), OXA (1 μM) or their combination with or without pretreatment of NAC in RKO (A) and LoVo (B) cells.

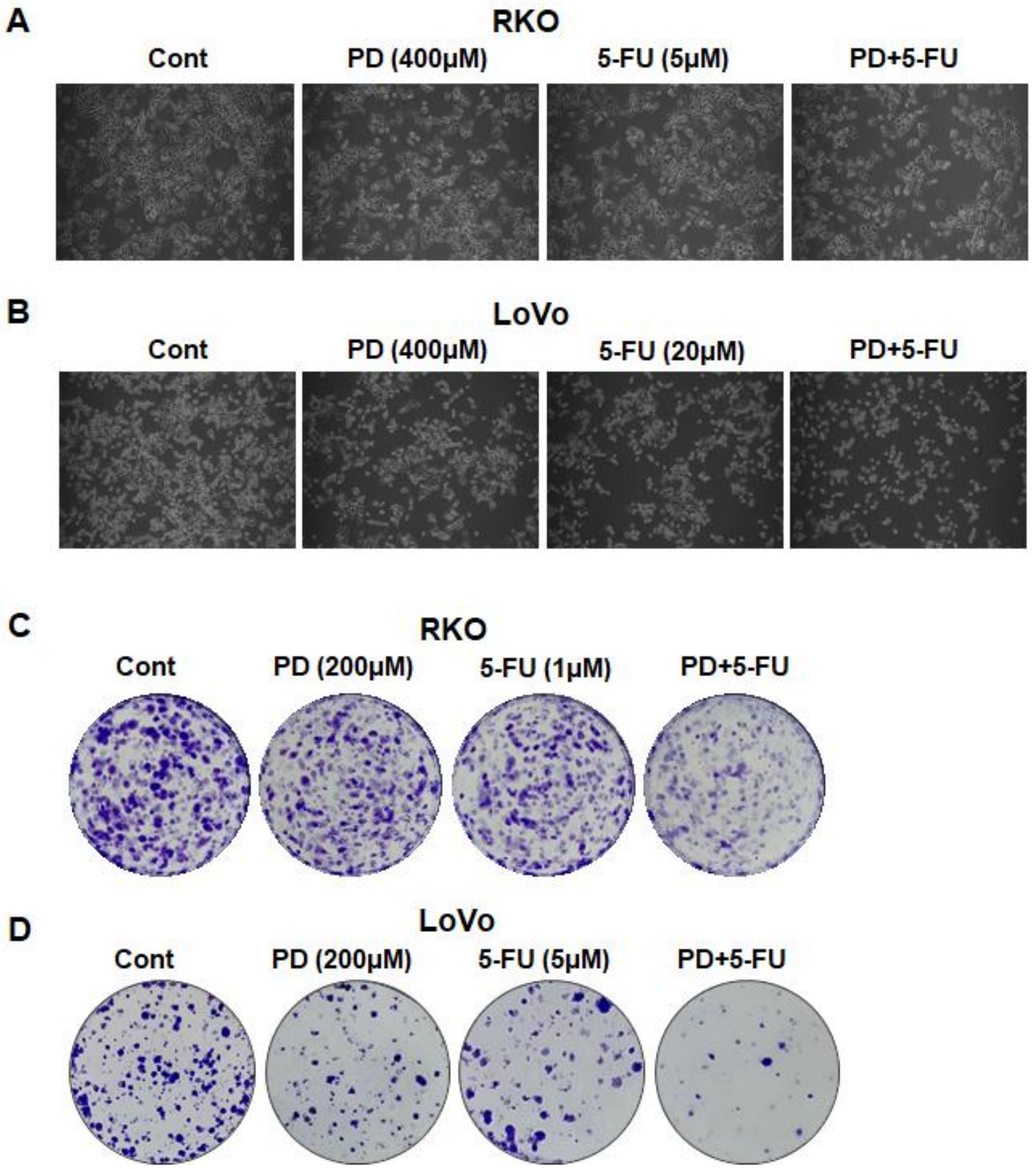

Figure S3. PD in combination with 5-FU exerted strong anti-tumor activity. (A-D) Cell viability (A, B) and colony forming ability (C, D) were measured following treatment with PD, 5-FU or combination theirof. Images were obtained at 100× magnification for cell viability assay.

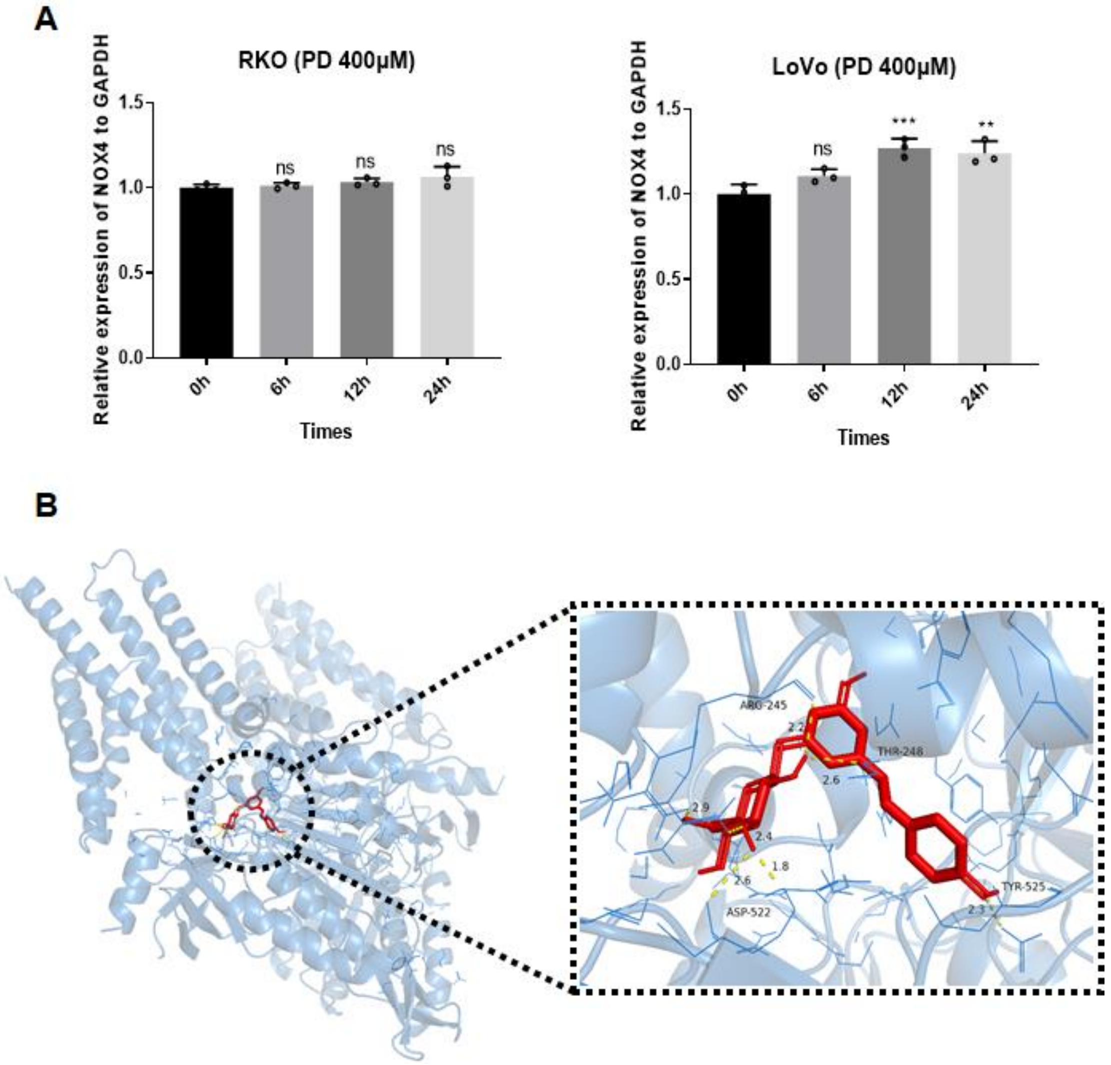

Figure S4. Molecular docking analysis. (A) The mRNA expression of NOX4 was measured by RT-qPCR after treatment with PD at different time points in RKO and LoVo cells. (B) Molecular docking results of PD and NOX5.

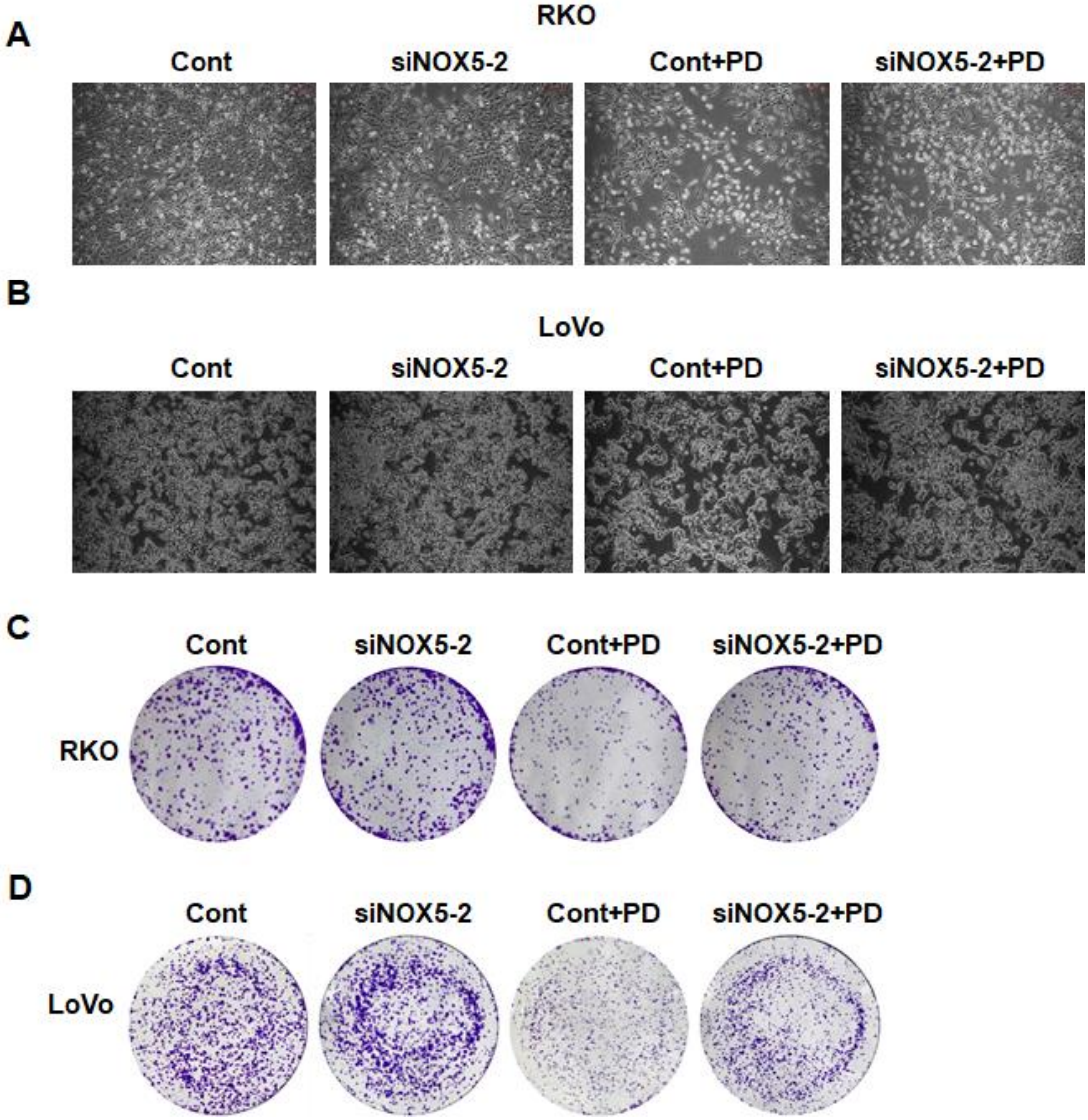

Figure S5. NOX5 knockdown attenuated anti-tumor activity of PD. (A-D) NOX5 knockdown colon cancer cells were treated with PD, and the cell viability (A, B) and colony forming ability (C, D) were assayed. Representative pictures were shown.

Table S1. Sequences of siRNA.

Name of siRNA	Sequences	
	Sense (5’-3’)	Antisense (5’-3’)
siNOX5-1	CCUUCUUUGCAGAGCGAUUTT	AAUCGCUCUGCAAAGAAGGTT
siNOX5-2	GCCUGGCAUUGGCUGGGUATT	UACCCAGCCAAUGCCAGGCTT
siCont	UUCUCCGAACGUGUCACGUTT	ACGUGACACGUUCGGAGAATT

Table S2. Sequences of RT-qPCR primers.

Genes	Primers	Sequences
NOX4	Forward	TGC TGT ATA ACC AAG GGC CA
	Reverse	CTG AGG CTC TGC TTA GAC AC A
NOX5	Forward	CCA GTG CCT CAA CTT CGA CT
	Reverse	GTA GGA CTT GAG CCA GCC AC
GAPDH	Forward	TCA AGG CTG AGA ACG GGA AG
	Reverse	GAC TCC ACG ACG TAC TCA GC
